# The Enjoyment of Knowledge Sharing: Impact of Altruism on Tacit Knowledge-Sharing Behavior

**DOI:** 10.3389/fpsyg.2020.01496

**Published:** 2020-07-16

**Authors:** Bojan Obrenovic, Du Jianguo, Diana Tsoy, Slobodan Obrenovic, Muhammad Aamir Shafique Khan, Farooq Anwar

**Affiliations:** ^1^School of Management, Jiangsu University, Zhenjiang, China; ^2^School of Media and Communication, Shanghai Jiao Tong University, Shanghai, China; ^3^Research and Development Department, Inovatus Usluge Ltd., Zagreb, Croatia; ^4^Lahore Business School, The University of Lahore, Lahore, Pakistan

**Keywords:** altruistic behaviors, impact of altruism on knowledge sharing, personality traits and knowledge sharing, tacit knowledge sharing, empirical study of behavior, willingness to share

## Abstract

Knowledge sharing between individuals is a key process for knowledge-intensive organizations to create value and gain a competitive edge. An individual is in the center of a complex set of factors, which are conducive to the knowledge-sharing process. The purpose of this empirical study is to explain the interaction mechanisms between personality and knowledge-sharing behavior and to examine the mediating effects of willingness to share knowledge and subjective norm. The theory of planned behavior, the social exchange theory, and the big five personality traits theory are combined to explain tacit knowledge-sharing behavior. A survey strategy and purposive sampling was applied, and the analysis was conducted on a sample of 288 employees from Croatia working on knowledge-intensive tasks for which high levels of tacit knowledge sharing are characteristic. A standard online questionnaire consisted of items evaluated on a 7-point Likert-scale, ranging from strongly agree (7) to strongly disagree (1). In the structural model, relationships between altruism, willingness, subjective norm, and tacit knowledge sharing were tested. Confirmatory factor analysis with maximum likelihood estimation was performed by using SEM software AMOS version 23. The findings of the study suggest that altruism has a direct impact on tacit knowledge sharing, reaffirming a relationship with knowledge sharing but distinguishing between sharing of different types of knowledge, assessing tacit knowledge sharing as a construct separate from general knowledge sharing. Our findings suggest that willingness to share is a predictive factor of knowledge sharing behavior between employees, having both direct impact on tacit knowledge sharing and being a mediator between the trait of altruism and tacit knowledge sharing. The mediation test also indicates that altruism has an indirect influence on tacit knowledge sharing when subjective norm was a mediator. The findings suggest that personality traits relying on social capital, such as altruism, have more influence on tacit knowledge sharing compared to personality traits that have accentuated intrinsic components. The study contributes to the better understanding of factors stimulating knowledge-sharing behaviors and provides recommendations based on empirical evidence, which may later be applied in the development of knowledge-sharing leadership styles, employee hiring, and auxiliary initiatives.

## Introduction

Knowledge-sharing behavior has a significant influence on team cohesion, working creativity, group performance, and the knowledge-integration process (Mesmer-Magnus and DeChurch, 2009; Obrenovic et al., 2015). Sharing of tacit and explicit knowledge leads to innovative ideas (Jackson and Knight, 2006) and improves task efficiency and organizational performance (Adenfelt, 2010). In addition, tacit knowledge sharing is especially valuable as it contributes to organizational productivity (Small and Sage, 2005; Reychav and Weisberg, 2009). The intention to share tacit knowledge depends on the various team and organizational determinants (Subramaniam and Venkatraman, 2001). Individual factors also play an essential role as tacit knowledge is closely related to a person’s experiences, thoughts, and beliefs (Alavi and Leidner, 2001). They shape how individuals interpret and construct knowledge. Diverse skills, experiences, and talents are crucial for bettering overall organizational performance; therefore, principal apprehension of individual motivation for knowledge sharing is vital for all managerial sciences.

Previous studies suggest that tacit knowledge sharing is grounded in individual characteristics (Matzler et al., 2008). For instance, the effects of group communication styles derived from personality traits impact knowledge-sharing behavior, which is mediated by knowledge-sharing attitudes of willingness and eagerness (De Vries et al., 2006). Additionally, traits of extraversion and agreeableness are positively associated with the desire to share knowledge (De Vries et al., 2006). Although the mentioned studies demonstrate a relationship between personality traits and knowledge-sharing behavior, many traits remain unexplored. A distinction between altruism and willingness is made to investigate to what extent these notions influence tacit knowledge sharing. Although in previous studies it has been posited that employees’ altruism and social interaction are crucial in promoting knowledge-sharing behavior (Ma and Chan, 2014; Wang and Hou, 2015; Sedighi et al., 2016; Carrera et al., 2018; Suwanti, 2019), how significant the mediation effect is and what prompts it have yet to be understood. In our study, we address this gap by utilizing constructs of altruism, subjective norm, and willingness to explain the knowledge-sharing behavior on the individual level, which can then be further generalized and applied in organizational settings. We combined the theory of planned behavior (Ajzen, 1991), the social exchange theory (Homans, 1958), and the big five personality traits theory (Goldberg, 1990) to explain the effect of personality traits on knowledge sharing and the mediating effect of willingness and subjective norm. Knowledge creation, compilation, enhancement, and transfer among individuals is a crucial process for knowledge-intensive organizations’ survival and competitiveness, and it relies almost exclusively on individuals’ consent and willingness to share it. Since individual knowledge sharing in business settings eventually results in positive performance outcomes, such as the company’s innovativeness, creativity, and flexibility, it has been a subject of many studies in knowledge management (Obrenovic and Qin, 2014). Literature suggests that it is simplistic to examine the behavior of a single organization in isolation as if it is a unique organism (Cummings and Thanem, 2002; Morgan, 2011; Faghih et al., 2016). As this paper suggests, an organization is rather a web consisting of influences, motivations, behaviors, actions, and reactions of diverse individuals and their dynamic interactions. Organizational and individual intentions interact in social settings, and this, in turn, fuels the knowledge-sharing behavior in working teams, contributes to team cohesion, and stimulates a sense of belonging. Although managerial literature points to activities that may contribute to employees’ willingness to share knowledge and comply with organizational norms (Wang et al., 2014; Safa et al., 2016), there is much more to learn about how specific strategies can be best applied to different personalities to elicit tacit knowledge sharing and encourage social bonding through individual-centric practices.

Therefore, a more comprehensive understanding of individual-level knowledge will contribute to psychology and the management science field. As Guldenmund (2010, p 13) pointed out, “The motor that drives the system to its desired end will always be particular idealistic individuals, not the system alone or the convictions it promulgates”. An individual is in the center of a complex set of factors, which either enable knowledge-sharing behavior or hinder it. To manage knowledge on a macro-organizational level, one must first understand what motivates and sways one to collaborate in sharing practice on the individual level. We examine the propensity of people to be altruistic and willing to share knowledge, focusing on personal, intangible, and tacit knowledge. The principal contribution of this paper is to render an empirical investigation on the levels of influences that stimulate knowledge-sharing behaviors and provide recommendations based on empirical evidence, which can be applied in the development of knowledge-sharing leadership styles and auxiliary initiatives.

Innovative points of the study translate to practical implications for managers and leaders alike, suggesting they should develop leadership styles that facilitate cultivation of social bonds and trust among employees, resulting in the willingness to share tacit knowledge. Knowing that altruistic individuals are predisposed to share knowledge willingly, managers can accordingly react and set specific roles for altruistic individuals within teams, assigning them tasks in which they would collaborate and engage in socialization with other people. Such decisions could enhance knowledge sharing in a group. Additionally, for employees with less altruistic characteristics, initiatives to prompt willingness, such as incentives and team activities that improve trust should be developed. The current study contributes to the existing occupational psychological and managerial literature, suggesting for future studies to consider other possible determinants relevant for knowledge sharing, such as personality traits of consciousness, neuroticism, and competitiveness, and test them on individuals working on less knowledge-intensive projects.

Altruism is a facet of agreeableness that represents a tendency of others to feel empathy; believe in a fair world; and be socially responsible, kind, and trusting of others (Bierhoff et al., 1991). Prior studies show that altruistic personality leads to organizational citizenship behavior (Organ, 1988). Caring and helping individuals relate to others and go beyond their regular job description to assist in solving tasks. Sharing knowledge might incite a feeling of pleasure and achievement (Kollock, 1999) among altruistic individuals as well as satisfy the desire to contribute to the working environment by helping others (Davenport and Prusak, 1998).

In prior studies, attitude and a subjective norm are used to explain knowledge sharing by applying knowledge-sharing intention as an indicator of knowledge-sharing behavior (Ryu et al., 2003; Chen et al., 2009). Even though individual characteristics have been acknowledged as a factor in knowledge sharing, a direct relationship between personality traits and attitude toward knowledge has been ignored for the most part. Therefore, the current study takes the individual trait of altruism and investigates its impact on subjective norm, willingness to share knowledge, and tacit knowledge sharing. Elements of the theory of planned behavior (Ajzen, 1991) are integrated into the research model, connecting them to an individual trait of altruism. Such model innovation represents a significant contribution of the study. Explaining the relationship between altruism and willingness to share knowledge in the context of knowledge sharing provides a better understanding of traits and attitudes that should be nurtured within individuals.

Expertise or specialization should be understood as emerging from continuous cyclic generation, gathering and compiling of tacit knowledge, and its affirmation in intergroup sharing of subjective know-how. The knowledge-sharing intensity varies depending on the industry type (Lin, 2007b), whereas effect of industry type in management research has been evidenced (Vij and Farooq, 2014). For instance, while tacit knowledge sharing is essential for innovation-oriented businesses to create additional value, explicit knowledge is more characteristic for manufacturing companies. Since we are interested in the dynamic motivational aspects of knowledge sharing, we purposely narrow our research to a sample of employees with a higher educational background. We assume higher schooling, training, and occupational residency advance knowledge-sharing practices and stress altruistic behavior, which are crucial for this research’s targeted industries. The success of innovation in these domains consists of knowledge enhancement and knowledge expanding through joint participation in a challenging environment and tasks, thus making employees more inclined to share their intellectual capital.

## Theory and Model

The theoretical framework is constructed based on the pillars of the theory of planned behavior, social exchange theory, and big five personality traits. The distinction between altruism, subjective norm, and willingness to share is made clear. We draw from the previous research of knowledge sharing built upon the theory of planned behavior (TPB), hypothesizing that intentions should lead us to the motivational components underlying the attitude (Mansor and Saparudin, 2015; Wu et al., 2015; Razak et al., 2016).

### Tacit Knowledge Sharing

According to Dyer and Nobeoka (2000), knowledge is the most potent weapon organizations possess, especially for knowledge-based companies. Organizations need to produce, collect, and share knowledge effectively (Zander and Kogut, 1995; Conner and Prahalad, 1996; Grant, 2013) since it encourages innovation and leads to higher organizational performance. Tacit knowledge exchange creates bonds and facilitates social communication between employees (Nonaka, 1994; Osterloh and Frey, 2000; Käser and Miles, 2002), leading to organizational success (Small and Sage, 2005; Reychav and Weisberg, 2009). Communication enables individuals to share and exchange knowledge and experiences, resulting in the development of expertise and complex skills. Consequently, collaboration and coordination between employees and team performance are enhanced by establishing a communicative environment (Marks et al., 2000). Besides, the group transactive memory system benefits from the knowledge shared (Moreland et al., 1996; Hollingshead, 1998a,b), which underpins the overall team accomplishment (Faraj and Sproull, 2000; Kanawattanachai and Yoo, 2002; Lewis, 2004).

Tacit knowledge was previously defined as experiential and intuitive in its nature (Faith and Seeam, 2018) and also subjective, context-specific, and difficult to capture (Razak et al., 2016), which makes it extremely valuable for organizational growth. Sharing of such knowledge is harder since it is not easily accessible and directly codifiable in formal language, but requires frequent face-to-face interactions. In group settings, employees working on joint tasks commonly store it in a “shared intellect” made up of members’ previous behaviors, insights, skills, responses, and behavioral models. Tacit knowledge is transmitted and gained exclusively due to internal motivations, such as socializing and during the social interaction (Lee and Choi, 2003). This process is described in terms of “learning by doing,” e.g., knowledge sharing develops during the interaction of employees with their coworkers and employees with the task at hand (Nonaka and Von Krogh, 2009). Not only is the socialization conducive to the sharing of technical skills and cognitive capital, but such collective learning also leads to the creation of new knowledge (Cabrera et al., 2006). Some studies characterize it in terms of action, experience, skills, and know-how embedded in professional collaboration (Alavi and Leidner, 2001; Göksel and Aydintan, 2017). It can be concluded that individuals with long tenure and intensive task involvement possess a significant amount of tacit knowledge since it is in its core embodied in action, commitment, and primarily derived from personal experiences that may be crucial for organizational survival and gaining a competitive edge. Previous empirical evidence stresses the moderating effect of employees’ personality traits on tacit knowledge sharing (Manaf et al., 2018).

### Personality and Attitude

In previous studies, big five personality traits and several facets are explored in the context of knowledge sharing. The link between personality traits and various attitudes has been confirmed (Francis et al., 1996; MacNicol et al., 2003). For instance, De Vries et al. (2006) confirms that willingness could mediate the effects of group communication styles derived from personality traits on knowledge-sharing behaviors.

Willingness to share knowledge was positively impacted by team members’ performance beliefs, job satisfaction, agreeableness, and extraversion. Additionally, extroverted team members, who had a strong faith in their performance and were satisfied with their job, possessed an attitude of eagerness to share knowledge (De Vries et al., 2006).

Altruism, which stems from the big five personality trait of agreeableness, is an attribute of individuals to be kind, caring, feel empathy, and engage in socially responsible behaviors (Bierhoff et al., 1991). Szuster (2016) defines altruism as manifested assistance that carries no costs in forging alliances, cooperation, and sharing something of value with others. She differentiates kin altruism (where the stakes can be as high as life or health) and reciprocal altruism demanding smaller sacrifices, being decidedly less spectacular. Altruistic people engage in helping strangers often at their own expense. For example, a knowledge worker sharing valuable knowledge, thus partially losing competitive advantage, can be considered pure altruistic behavior.

Contrary to the early notions of altruism, which found it to be theoretically improbable due to the nature of all behavior to be understood in terms of self-interest, new socioeconomic perspectives prompted the understanding as coming from within the benefits and costs framework. The sociological construct of altruism is, thus, relatively new and, in general, deals with investigating what leads people to act selflessly. Accordingly, employee altruism may be defined in terms of intentional, voluntary behavior aimed at improving another coworker’s condition, skill, or knowledge without reciprocal expectancy or personal gain. Bkeikher et al. (2016) posited there are three dominant approaches to the concept of altruism, namely altruism as a form of evolutionary self-preservation, as individual psychological motivation to do good, and as a shared moral norm for behavior. Altruistic prosocial behaviors are a voluntary conduct stimulated by internalized norms and sympathy for the welfare of others (Carlo and Randall, 2002). This behavioral trait is further argued for on the grounds of heritability of sympathy, adolescent stabile display of prosocial behavior, and the existence of altruism in a variety of contexts. As sympathy allows one to respond by taking another’s perspective, it may incite one’s desire to relieve others of distress. Altruistic tendency is found to be positively related to perspective-taking and internalized prosocial reasoning. In the organizational setting, it fits perfectly into a context of knowledge sharing by granting coworkers help and expertise necessary to solve tasks at hand (Carlo and Randall, 2002). Furthermore, due to the internalized norms foundation, altruism is found to be a higher-order moral reasoning based on the principle to help others, and it is often hypothesized altruistic individuals will more likely engage in behavior corresponding to the norm.

The current study approaches altruism as a psychological motivation to exhibit selfless and community-beneficial behavior arising from the enjoyment in knowledge sharing. Altruistic employees perceive participating in knowledge sharing as contributing to a group to which an individual feels strongly connected, whether it is represented in one’s coworkers, superiors, or organization as a community-based partner. Knowledge sharing may be motivated by the desire to help others (Davenport and Prusak, 1998) and is experienced as a sense of fulfillment and enjoyment from when the action is attained (Kollock, 1999). Consequently, altruistic individuals feel gratification when they engage in helping behaviors. They exhibit a positive perception and feeling toward collectivism. Therefore, the concept of enjoyment of helping others has been derived from altruism and was used in prior studies to measure altruism.

Altruism has been explored within the context of explicit knowledge sharing (Wasko and Faraj, 2005; Wu et al., 2009). Chang and Chuang (2011) show that altruism increases the quality and quantity of knowledge sharing in virtual communities. Moreover, altruism makes a significant contribution to the willingness to continue sharing knowledge among the members of such communities (Zhang et al., 2010). As for the enjoyment component of altruism, it affects the quality of knowledge contribution (Wasko and Faraj, 2005) and willingness to donate and collect knowledge (Lin, 2007c). Altruistic employees often go beyond their regular job responsibilities and engage in organizational citizenship behavior, such as assisting their colleagues with job tasks. IT knowledge workers share information and know-how through the knowledge management system (KMS), consequently feeling gratification from helping others (He and Wei, 2009). Also, sharing behavior and positive attitude toward knowledge sharing improves as satisfaction in assisting others to increases (Lin, 2007b).

Prior studies find knowledge sharing to be highly influenced by individuals’ enjoyment in helping others and relies heavily on altruistic personality traits (Kankanhalli et al., 2005; He and Wei, 2009; Huang et al., 2011). Specific authors have defined altruism as a selfless voluntary attempt to help others with the aim of improving the community welfare even at one’s cost (Fang and Chiu, 2010). In line with their findings and consistent with TPB, altruism was found to be the antecedent to knowledge-sharing intention, contending that altruism is positively associated with knowledge-sharing continuance intentions. Intrinsic motivation is defined as knowledge sharing for its inherent satisfactions rather than for tangible rewards, and altruism represents such an intrinsic motivation contingent upon the perception of gratification from helping coworkers (Sedighi et al., 2016). Employees who are intrinsically motivated find the activity of exchange to be exciting and fun, so they often enjoy disseminating knowledge, and in that regard, they are inclined to take on challenging and complex tasks. In doing so, they contribute to self and organizational interests (Lin, 2007a; Minbaeva et al., 2009).

Altruism is an especially relevant behavioral trait to consider when choosing a team in innovation-driven industries since it was found to reduce relationship conflict and enhance participative processes (Eddleston and Kellermanns, 2007). This is of great significance when considering that knowledge sharing may be rendered as a communication process, and its viability is determined by the willingness of all parties to interact and help each other with regards to the volume and quality of knowledge (Lou et al., 2013). If there are any interruptions, communication may break down, thus making knowledge transfer unlikely. The facet of altruism and prosocial behaviors stemming from this trait may grant such cohesion, especially in cases where a monetary reward system may not be sufficient to encourage knowledge transmission. Furthermore, from a cognitive point of view, when individuals are intrinsically motivated by their altruistic nature, the stimulation of positive creativity enables them to access more information and simplify the identification of ideas in a more flexible way (Suwanti, 2019). The association was also made between trust and altruism. While the self-concerned motivation for knowledge sharing relies on mutual trust among knowledge sharers, for they expect potential returns from knowledge recipients in the future, in the case of altruism, such reciprocity is less important. Taking into account that altruistic individuals act on altruistic motivation, interpersonal trust is not vital. Wu et al. (2009) find that the effect of interpersonal trust is more significant for employees low in altruism than for highly altruistic employees; thus, the trait of altruism reduces the positive association between trust of colleagues and knowledge sharing.

Considering the great significance of personality traits in literature and in explaining social behavior in the workplace (Matzler and Mueller, 2011), an individual characteristic of altruism has been introduced as an antecedent of tacit knowledge sharing. People who exhibit altruistic attributes in the sense of enjoyment while helping others are more inclined to share their knowledge as it pleases them, and they do so primarily due to their positive feelings toward a collective.

As a result, the following hypothesis is derived:

**H1:** There is a positive impact of altruism on willingness to share knowledge.

### Willingness to Share Knowledge

Willingness refers to “the extent to which an individual is prepared to grant other group members access to his intellectual capital” (van den Hooff et al., 2012). Willingness originates from emphasizing the collective’s interests (De Vries et al., 2006), i.e., reflecting a favorable attitude toward group members. The philosophy of willingness primarily considers the members of the group to be the most relevant factor leading to intention formation and behavior performance. The concept of reciprocity is in the essence of willingness, expecting others also to contribute knowledge (Nahapiet and Ghoshal, 1998; Adler and Kwon, 2002).

We may characterize willingness as the extent to which individuals are ready to transfer their intellectual resources and provide access to personal knowledge they have acquired to other team and organizational members. As opposed to eager employees, willing individuals are considered as having low internal drive to share their knowledge with others and, therefore, should be motivated externally (Van den Hooff and Hendrix, 2004; Ziemba and Eisenbardt, 2014). In their conditional way, they are susceptible to incentives; e.g., their motivation stems from expected reciprocity, monetary and non-monetary awards, and prestige.

By the social exchange theory (SET), willingness to transfer knowledge is high under the expectancy of reciprocal benefits. Mutual exchange happens in trusting relationships with coworkers built over time. Whereas this reciprocity proves to be beneficial to employees, it also becomes useful to the organization (Tohidinia and Mosakhani, 2010; Krok, 2013; Kuo, 2013; Loebbecke et al., 2016). For instance, the reciprocal relationship between the protégé and mentor builds the relationship between the protégé and the organization (Curtis and Taylor, 2018). Knowledge collecting is very indicative, where eager employees actively participate in the process of donating, willing employees are rather reactive in their behavior. Employees are frequently reluctant to share their knowledge due to the fear of losing an edge (Kankanhalli et al., 2005; Alsharo et al., 2017). When trying to stimulate employees’ willingness and innovative behaviors, perceived organizational support has a crucial role as it increases the effect of trust and encourages knowledge sharing (Kambey et al., 2018; Le and Lei, 2019). Not only does the willingness to share knowledge stem from relationships with coworkers, but according to Malik and Kanwal (2018), so does their level of job satisfaction and performance affect their motivation and willingness to contribute; i.e., the higher the level, the stronger the willingness to donate knowledge. Motivation to share is triggered by intrinsic and extrinsic rewards and perception of knowledge value (McNichols, 2010).

Susanty et al. (2016) point out that personal intention to share information is the fundamental determinant for knowledge sharing. Given that willing people have no internal drive to share knowledge but are more passive and reactive, they expect others to contribute knowledge. Subsequently, the balance between knowledge donating and collecting is a target objective of willing people. In a study conducted by De Vries et al. (2006), it was confirmed that willingness is linked to knowledge collecting and knowledge donating. The knowledge-sharing attitude of willingness mediates communication styles, job satisfaction, and performance beliefs’ relationship to knowledge-sharing behavior. Additionally, willingness is influenced by emotions and serves as a mediator between pride, empathy, and intention to perform an action (De Vries et al., 2006).

In line with the basic principle of the theory of planned behavior and the extensive empirical evidence to support this proposition, we conclude that the attitude of willingness affects knowledge-sharing behavior. Therefore, we find that willingness to share knowledge influences tacit knowledge sharing and serves as a mediator between knowledge-sharing behavior and personality characteristics. When planning the behavior, alternative choices are analyzed in order to identify the one that will most likely lead to the desired goal. When individuals perceive that joint effort makes the completion of the targeted task more probable, they will willingly share their experience with others under the condition of reciprocity; e.g., they will share knowledge in order to gain knowledge necessary to complete the task. Therefore, their willingness to exert the sharing behavior increases when there is a visible relationship between effort and the result, namely when individuals perceive that joint effort makes the result plausible.

**H2**: There is a positive impact of willingness to share knowledge on tacit knowledge-sharing behavior.

### Subjective Norm

Subjective norm refers to the reactions or thoughts and sometimes social pressure that arises in regards to a particular action, represented by the circle of people involving family, neighbors, colleagues, or other relevant members of the community. Such reactions might help to determine or assure that the behavior conforms to the rules of society (Fishbein and Ajzen, 1975, 1981). The relevant others who would react to the individual’s activities are often displaying some model behavior with which one may desire to comply with feeling like a part of a group (Pavlou and Fygenson, 2006). The subjective norm mainly determines whether the relevant others would accept the action (Thompson et al., 1991; Taylor and Todd, 1995; Strauss et al., 2000; Venkatesh et al., 2003). The subjective norm has two components: one is some normative ideas or judgments of the society, and the other is the individual’s desire to accommodate them (Fishbein and Ajzen, 1975, 1981). Following the theory of the need to belong (Baumeister and Leary, 1995), people tend to engage in socializing to attain a sense of belonging. This theory accounts for the motivation to affiliate. As cognitive exchange occurs during social interactions, individuals may take part in knowledge sharing in order to bond with others, or they may comply with the subjective norm to be accepted. While people are inclined to fulfill different needs, a sense of belonging is considered to be one of the most basic and even innate.

Altruistic individuals are receptive to the needs of others, consider the expectations of relevant others, and tend to seek their insight. Due to this outward orientation, they comply with existing social norms. More particularly, altruistic individuals tend to share knowledge with other members when a team norm instructs it. Therefore, we conclude the following:

**H3:** There is a positive impact of altruism on subjective norm toward knowledge sharing.

Past research shows that subjective norm has an impact on behavioral intentions toward system use (Karahanna et al., 1999) and indirectly affects the use in virtual learning communities (Van Raaij and Schepers, 2008). In a study of 1027 organizational leaders and collaborators, the relationship between knowledge sharing and subjective norm was found, suggesting that an employee’s attitude is shaped by the demand to share knowledge coming from the people of significance in the workplace (Castañeda and Ignacio, 2015). The study conducted by Bock et al. (2005) indicated that organizational environment, external motivators, and psychosocial factors influence team members’ knowledge-sharing patterns and willingness by shaping normative beliefs and opinions (Bock et al., 2005). Such patterns and intentions as well as their effect on the usage in learning communities (Karahanna et al., 1999) and systems, significantly depend on the subjective norm (Van Raaij and Schepers, 2008). Based on the theoretical support and empirical evidence on the relationship between subjective norm and behavior, we posit that subjective norm affects the sharing of tacit knowledge between employees.

**H4:** Subjective norm toward knowledge sharing has a positive impact on tacit knowledge sharing.

### Mediating Effects

In a mediation model, willingness and subjective norm mediate the relationship between altruism and tacit knowledge sharing. The above-reviewed literature using the theory of planned behavior, social exchange theory, and personality traits theory provides a strong justification for the mediating model, supporting individual relationships between variables of the model. The theory of planned behavior is used to explain the effect of personality traits on knowledge sharing and the mediating effect of willingness and subjective norm. Big five personality traits model and social exchange theory explain the interaction of altruism as a facet of agreeableness (Goldberg, 1990) with willingness and knowledge sharing. According to the big five personality model, the agreeableness trait reflects individual concern for social harmony, and such individuals are characterized as altruistic, trusting, helpful, and cooperative. Altruism was positively correlated with quality relationships within a workplace, and some academics speculate that organizations may be able to determine an individual’s future performance based on this trait (Sackett and Walmsley, 2014). In prior studies, the theory of planned behavior was acknowledged as the most influential theory of human behavior. Attitude and subjective norm were used to explain knowledge-sharing intention as an indicator of knowledge-sharing behavior (Ryu et al., 2003; Chen et al., 2009). When individuals perceive that the subjective norm supports knowledge-sharing behavior, they are more willing to comply with it, and a favorable attitude toward a behavior together with a positive subjective norm forms an individual’s intention to engage in the behavior. Normative beliefs regarding the expectation of relevant others, be it coworkers, friends, or superiors, shape the attitude and affect the intention to engage in sharing activity. According to the social exchange theory, social behavior involves social exchanges where individuals are motivated to attain a reward for which they must forfeit something of value (Redmond, 2015). Therefore, knowledge transfer is a voluntary process between a knowledge source and knowledge recipient. People are willing to disseminate and communicate their knowledge, in both explicit and tacit form and help to correct problems and generate new ideas when they anticipate, in line with expectancy value theory, to gain monetary or non-monetary reward in return, such as bonuses, promotion, and recognition. Central here is the notion of reciprocity. Therefore, we conclude the following:

**H5**: There is a mediating effect of willingness and subjective norm between altruism and tacit knowledge sharing.

## Materials and Methods

### Participants and Procedure

The purposive sampling technique was applied, and a survey strategy was employed. Data were collected from the employees of companies based in Croatia. The study participants were employees of technology companies, working mainly on semiconductors, electronics, medicine, chemistry, IT, photonics, and biochemistry. The employees were working on challenging and knowledge-intensive tasks, for which high levels of tacit knowledge sharing are characteristic. A standard online survey was sent out in an email to 600 participants. A total of 432 respondents filled out the questionnaire. After eliminating missing values, a final sample consisted of 288 employees. Most of the subjects had a Ph.D. degree (89%), a master’s degree (9%), and more than 20 years of work experience (36%). Male participants were, by and large, over 50 years old. To ensure the validity and reliability of the measurement tool, the scales were modified to suit the context of the research and checked for face validity. Team-level and intrapersonal perspective items were removed or rewritten to suit the context of the individual-level tacit knowledge-sharing behavior. Upon reviewing the questionnaire items, slight modifications were made in vocabulary and logic. The example items are reported in the measurement section. The questionnaire was administered in the English language. All the items were evaluated on a 7-point Likert-scale ranging from strongly agree (7) to strongly disagree (1). Data on tacit knowledge-sharing behavior, willingness to share knowledge, subjective norm, and altruism were collected. Additionally, demographic data, gender, age, education, tenure, and industry type were collected. Construct definitions, key references, number of items for each construct, and Cronbach’s alpha, α, and McDonald’s omega, ω, are displayed in Table 1.

**TABLE 1:** Construct definitions and reliability values.

Constructs	Definitions	Key references	Items	Measurement
Tacit knowledge sharing	The extent to which employees collect and share know-how, know-where, know-who, expertise, and lessons learned from past failures	Wang and Wang, 2012	7	α = 0.97
				ω = 0.97
Subjective norm	The extent to which one believes that people who bear pressure on one’s actions expect one to perform the behavior in question multiplied by the degree of one’s compliance with each of one’s referents	Fishbein and Ajzen, 1975, 1981; Bock et al., 2005	3	α = 0.82
				ω = 0.83
Willingness	The extent to which an individual is prepared to grant other group members access to their intellectual capital and expertise	Bouwman et al., 2005; van den Hooff et al., 2012	3	α = 0.80
				ω = 0.81
Altruism	To the extent to which employees enjoy helping others voluntarily and intentionally	Wasko and Faraj, 2000; Lin, 2007c	4	α = 0.91
				ω = 0.92

### Measurements

#### Tacit Knowledge Sharing

To assess the tacit knowledge-sharing behavior, the items were adopted from Wang and Wang (2012), who developed the scale by combining items from past studies (Bock et al., 2005; Lin, 2007a,b,c; Holste and Fields, 2010; Reychav and Weisberg, 2010). Their scale indicated an acceptable Cronbach’s α value (α = 0.97). We modified the items to make the assessment consistent with the contexts of knowledge sharing on the individual level and communication toward other employees. Used items refer to sharing and collecting the individuals’ experience, know-where, and know-who expertise and lessons learned. Example items include “I frequently share knowledge based on my experience” and “I frequently share knowledge of know-where or know-whom with colleagues.”

#### Willingness to Share Knowledge

Willingness to share knowledge was measured with the three-item scale adopted from the work of De Vries et al. (2006). The willingness of an individual to share expertise and individual intellectual capital with the other team members was estimated with a scale of adequate reliability (α = 0.80). The items assessed improving colleagues’ performance and collaboration by sharing knowledge and expectations of colleagues sharing their knowledge after they had received help. Example items are “I try to improve my colleagues’ performance by sharing knowledge” and “I think that sharing my knowledge contributes to improved collaboration with my colleagues.”

#### Subjective Norm

The subjective norm scale was adopted from the work of Bock et al. (2005), who based their scale on the research of Fishbein and Ajzen (1975). Normative beliefs of project team members’ workmates, supervisors, and project managers regarding sharing knowledge were measured using a three-item scale. Example items include, “My immediate supervisor thinks that I should share my knowledge with my colleagues” and, “My colleagues think that I should share my knowledge with them.” Cronbach’s α of the scale used to evaluate the internal consistency was acceptable (α = 0.82).

#### Altruism (Enjoyment in Helping Others)

Altruism was evaluated by applying Lin’s four-item scale Lin (2007c), which he obtained from Wasko and Faraj’s research Wasko and Faraj (2000). In these studies, altruism was conceptualized as the feeling of satisfaction in helping others and had concentrated on the sense of enjoyment employees felt while they were sharing knowledge (α = 0.91). The example items are “I enjoy sharing my knowledge with colleagues” and “It feels good to share my knowledge with someone else.”

### Statistical Analysis

The analysis was conducted on a sample of 288 individuals from companies in Croatia. Preliminary analysis included descriptive statistics calculation, reliability, validity, and normality checks. Descriptive statistics indicators include mean, which indicates a central tendency of the data set and standard deviations, standard errors, kurtosis, and skewness, all indicating variability (Table 2). Shapiro–Wilk and Kolmogorov–Smirnov tests were used to determine normality of the data set. The Shapiro–Wilk test can be considered as fitting for sample sizes as large as 2000 and has the highest power for a given significance (Razali and Wah, 2011). Reliability and validity tests of the measurement tool were performed using SPSS, indicating appropriateness. Discriminant validity was measured with MSV < AVE and square root of AVE greater than interconstruct correlations, thus satisfying conditions.

**TABLE 2:** Descriptive statistics.

Statistic	Mean	*SD*	Kurtosis	Skewness
Tacit knowledge sharing	5.76	0.97	1.58	−0.90
Willingness	6.01	0.70	1.59	−0.92
Subjective norm	5.59	0.82	1.08	−0.78
Altruism	6.16	0.64	1.01	−0.68
*N* = 288				

**FIGURE 1:**
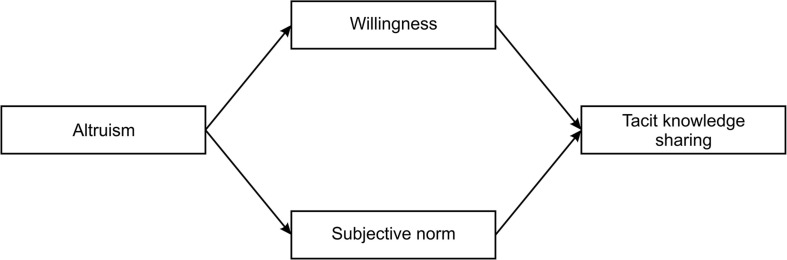
Research model of the study.

**FIGURE 2:**
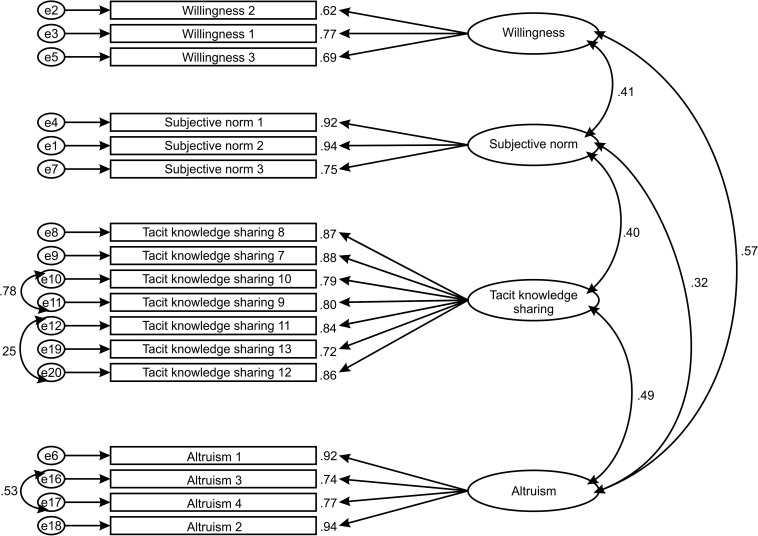
Measurement model.

Confirmatory factor analysis with maximum likelihood estimation was performed in SEM software AMOS version 23. This study was conducted following a two-step analytical strategy to test the hypothesized model (Figure 1) as recommended by Anderson and Gerbing (1988). Accordingly, the measurement model was tested independently before testing and evaluation of the full structural model (Medsker et al., 1994). The confirmatory factor analysis was performed. The four-factor model was hypothesized in measurement model testing (Figure 2). To assess the model fit, goodness of fit indices χ^2^/df, CFI, SRMR, and RMSEA were calculated. Next, the relationships between altruism, willingness, subjective norm, and tacit knowledge were tested as a part of the structural model assessment. Additionally, to provide complete path analysis, standardized parameter estimates, standard errors, and *p*-values for the structural model were calculated. The significance level was set at *p* < 0.001. The judgment of a good model fit was done with 1.96 < C.R. < -1.96. Mediation effects were tested by applying a bootstrap approach and examining the changes in the relationship between altruism and tacit knowledge after introducing the willingness and subjective norm as mediators. The significance of the relationship was assessed with a *p*-value at the significance levels from 0.05 to 0.001.

## Results

### Results of Confirmatory Factor Analysis and Path Analysis

Results of the normality tests indicate that data is normally distributed. The null hypothesis is that data fits the normal distribution. Since all the statistic values for the Kolmogorov–Smirnov and Shapiro–Wilk tests are higher than the level of significance of 0.05, 0.894 for willingness, the subjective norm of 0.956, 0.886 altruism, and 0.921 tacit knowledge sharing in the Shapiro–Wilk test, we cannot reject the null hypothesis and can conclude that data fits the normal distribution reasonably well for all variables in the model. To verify the data fit, normality was additionally assessed using QQ’ plots confirming data fitness.

The results of the confirmatory factor analysis revealed that the measurement model was not a good fit (χ^2^/df = 5.312; CFI = 0.875; SRMR = 0.065; RMSEA = 0.123). Therefore, to achieve a better fit, we proceeded to covary item errors given that certain items share similarities in their wording, a practice justified by Byrne (2010). Thus, guided by modification indices with values higher than 20, covariances were added to two sets of item errors of altruism to improve the model fit. Additionally, covariances were added to two sets of item errors of tacit knowledge due to reverse wording in the questionnaire. Covariance of reverse-worded items is justified by Brown and Moore (2012).

After covarying within-item errors, the improved measurement model was tested again and achieved a good model fit. Considering that the chi-square test has been criticized for its sensitivity to sample size (Wheaton et al., 1977; Byrne, 2010), alternative goodness of fit indices (χ^2^/df, GFI, SRMR, RMSEA, and CFI) are used in this study. Thus, model adequacy was assessed with population discrepancy function and represented by root mean square error of approximation (RMSEA = 0.071). It is higher than 0.05; however, it is less than the threshold advised by Ferdinand (2002) of 0.08 that is interpreted as an acceptable level of model fit. Goodness of fit is also justified by Cangur and Ercan (2015) if the RMSEA falls in the range of 0.05 and 0.08. However, it is advisable to refer to the comparative fit index (CFA) when sample size is small (Hu and Bentler, 1998; Chen, 2007). The statistic CFI = 0.961, which is above the recommended 0.95, indicates excellent model fit. As for the other indices, χ^2^/degrees of freedom ratio (χ^2^/df = 2.396) was below the recommended value of 3, representing an excellent fit (Hair et al., 2013), the standardized root mean square (SRMR = 0.058) was less than 0.08, also indicating a good model fit (Byrne, 2010). Obtained results indicated that the model achieved a good model fit (Hu and Bentler, 1999; Byrne, 2010). Table 3 provides the comparison of model fit indices after improving the measurement model.

**TABLE 3:** Comparison of model fit indices.

	χ^2^/df	SRMR	RMSEA	CFI
Measurement model	5.312	0.065	0.123	0.875
Improved measurement model	2.466	0.058	0.071	0.961
Thresholds*	1 < df < 3	<0.08	<0.06/10	>0.90/95

χ^2^/df, normed chi-square statistic; GFI, Goodness-of-Fit Index; RMR, Root-Mean-Square Residual; RMSEA, Root Mean Square Error of Approximation; NFI, Normed Fit Index; TLI, Tucker-Lewis Index; CFI, Comparative Fit Index. *References: Hu and Bentler (1999), Hoyle (2000), Thompson (2005), and Kline (2011).

Unstandardized and standardized parameter estimates obtained in this research are provided in Table 4. Considering the results, it can be concluded that unstandardized estimates (Table 4) are all statistically significant given C.R. values are above the recommended threshold (1.96).

**TABLE 4:** Standardized parameter estimates, standard errors, and *p*-values for the measurement model.

			Estimate	SE	C.R.	*P*
WIL2	<–	WILL	1.000			
WIL1	<–	WILL	1.028	0.117	8.797	***
WIL3	<–	WILL	0.782	0.092	8.489	***
EKS8	<–	TACIT	1.000			
EKS7	<–	TACIT	0.996	0.055	18.134	***
EKS10	<–	TACIT	1.029	0.062	16.562	***
EKS9	<–	TACIT	1.053	0.063	16.827	***
EKS11	<–	TACIT	0.945	0.052	18.202	***
EHO3	<–	EHO	1.000			
EHO4	<–	EHO	1.287	0.066	19.480	***
EHO2	<–	EHO	1.351	0.083	16.189	***
EKS13	<–	TACIT	1.041	0.073	14.341	***
EKS12	<–	TACIT	1.004	0.053	19.080	***
EHO1	<–	EHO	1.353	0.085	15.922	***
NBKS1	<–	NBKS	1.000			
NBKS2	<–	NBKS	1.003	0.042	23.910	***
NBKS3	<–	NBKS	0.763	0.047	16.344	***

***p < 0.001; SRW, Standardized Regression Weights.

**FIGURE 3:**
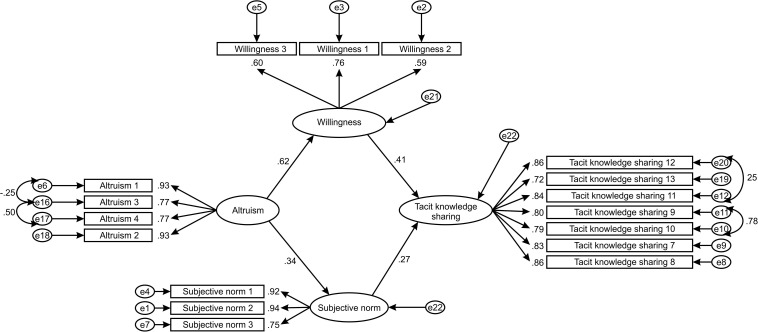
Hypothesized structural model.

After the confirmatory factor analysis, the path analysis in SEM was conducted, and the structural model was tested. The hypothesized model (Figure 3) was tested for the model fit. RMSEA with 0.08 < 0.076 < 0.05 was justified by Ferdinand (2002) and Cangur and Ercan (2015) as an acceptable level of fit. Referring to Hu and Bentler (1998) and Chen (2007), for a sample size of 288, CFI is a relevant measure. CFI = 0.953 was above the threshold of 0.95, indicating an excellent model fit. The indicators CMIN/DF = 2.637 and SRMR = 0.088 also fulfill the threshold requirements, reaching acceptable and excellent levels, respectively. Based on these assessments, a good model fit of the structural model can be concluded. The results are given in Table 5.

**TABLE 5:** Summary of model fit indices.

	χ^2^/df	SRMR	RMSEA	CFI
Hypothesized structural model	2.637	0.088	0.076	0.953
Thresholds*	<3	<0.06/10	<0.08	>0.90/95

χ^2^/df, normed chi-square statistic; GFI, Goodness-of-Fit Index; RMR, Root-Mean-Square Residual; RMSEA, Root Mean Square Error of Approximation; NFI, Normed Fit Index; TLI, Tucker-Lewis Index; CFI, Comparative Fit Index. *References: Hu and Bentler (1999), Hoyle (2000), Thompson (2005), and Kline (2011).

The results listed in Table 5 indicate all direct paths in the structural model were statistically significant. The substantial direct impact was between altruism–enjoyment in helping others (EHO) and willingness (WILL) (β = 0.619; *p* < 0.001). Further, willingness has a positive moderate direct effect on tacit knowledge sharing (TACIT) (β = 0.411; *p* < 0.001) while the subjective norm (NBKS) has a positive weak direct effect on tacit knowledge sharing (β = 0.272; *p* < 0.001). Last, the altruism-enjoyment in helping others has a positive weak direct effect on NBKS (β = 0.338; *p* < 0.001). Consequently, all the hypothesized relationships of the study were accepted (Table 6).

**TABLE 6:** Standardized parameter estimates, standard errors, and *p*-values for the structural model.

			SRW	USRW	*SE*	C.R.	*P*
NBKS	<–	EHO	0.338	0.732	0.136	5.402	***
WILL	<–	EHO	0.619	0.755	0.106	7.127	***
TACIT	<–	WILL	0.411	0.588	0.107	5.497	***
TACIT	<–	NBKS	0.272	0.219	0.047	4.638	***
WIL2	<–	WILL	0.594	1			
WIL1	<–	WILL	0.762	1.072	0.126	8.485	***
WIL3	<–	WILL	0.687	0.817	0.1	8.172	***
EKS8	<–	TACIT	0.863	1			
EKS7	<–	TACIT	0.83	0.995	0.056	17.857	***
EKS10	<–	TACIT	0.788	1.031	0.063	16.361	***
EKS9	<–	TACIT	0.795	1.054	0.063	16.61	***
EKS11	<–	TACIT	0.835	0.943	0.053	17.832	***
EHO3	<–	EHO	0.768	1			
EHO4	<–	EHO	0.773	1.239	0.064	19.204	***
EHO2	<–	EHO	0.93	1.287	0.078	16.417	***
EKS13	<–	TACIT	0.716	1.042	0.074	14.143	***
EKS12	<–	TACIT	0.859	1.004	0.053	18.765	***
EHO1	<–	EHO	0.927	1.319	0.085	15.601	***
NBKS1	<–	NBKS	0.919	1			
NBKS2	<–	NBKS	0.945	1.014	0.043	23.72	***
NBKS3	<–	NBKS	0.746	0.764	0.047	16.214	***

***p < 0.001; SRW, Standardized Regression Weights; URW, Unstandardized Regression Weights; C.R., critical value.

### Mediating Effects

Along with the establishment of direct relationships, the mediating effects were tested. Mediating effects of willingness and subjective norm between the independent variable of altruism and the dependent variable of tacit knowledge sharing are tested. To calculate mediating effects, a bootstrap approach was used, and 95% of the bias-corrected bootstrap confidence interval (CI) of the unstandardized indirect effects were applied. As the first step, willingness was included as the mediator in the relationship between altruism and tacit knowledge sharing. Referring to Table 7, willingness mediates the relationship between altruism and tacit knowledge sharing, which is confirmed by a positive value of β = 0.149 and bootstrap test (*p* < 0.05). The role of subjective norm as a mediator was also determined. Similarly to willingness, its impact on the relationship between altruism and tacit knowledge sharing is positive. The mediating effect of subjective norm (β = 0.088) is less than the value of the direct relationship of altruism and tacit knowledge (β = 0.403), but the bootstrap test indicates the same level of significance with *p* = 0.01, thus, confirming the Hypothesis 5.

**TABLE 7:** Direct, indirect, and total effects.

Pathway	Standardized direct effect	Standardized indirect effect	Standardized total effect	Evidence of
Tacit knowledge sharing <– Altruism	0.344**	0.149*	0.493**	Indirect positive, significant effect (Willingness as mediator)
Tacit knowledge sharing <– Altruism	0.403**	0.088**	0.491**	Indirect positive, significant effect (Subjective norm as mediator)

*p-value 0.050; **p-value 0.010.

## Discussion

The study examined factors impacting individual knowledge-sharing behavior. Current empirical research has used prior knowledge (Kogut and Zander, 1993; Bock et al., 2005; Füller et al., 2014) to create an innovative tacit knowledge-sharing model, consequently enriching the field of organizational psychology. We examined the impact of the personality trait of altruism, conceptualized as enjoyment in knowledge sharing, on tacit knowledge sharing and the mediating effect of willingness and subjective norm. In line with the big five personality traits model, altruistic individuals are characterized as trusting, helpful, cooperative, and due to their gentle nature, more inclined to comply with the subjective norm to maintain social harmony and attain a sense of belonging. They share knowledge to increase the community welfare and expect little or nothing in return. The theory of planned behavior was partially applied to account for attitude and subjective norm in forming knowledge-sharing behavior (Sedighi et al., 2016; Suwanti, 2019). Furthermore, we discovered that the altruistic trait presupposes a higher subjective norm to share knowledge. Altruistic individuals are oriented outward, considerate, and pay attention to the needs of others. They share knowledge when a norm instructs it. More precisely, when individuals perceive that subjective norm supports knowledge-sharing behavior, they are more willing to comply with it, and a favorable attitude toward a behavior together with a positive subjective norm form an individual’s behavior. Altruistic individuals take part in knowledge sharing to bond with others, and they comply to the subjective norm because of their underlying innate need to be accepted (Lou et al., 2013).

The study findings suggest that altruistic individuals experiencing enjoyment in helping others are more willing to share knowledge. Individuals motivated intrinsically by the satisfaction that they get from assisting colleagues exhibit a more positive attitude toward the collective and are more willing to share knowledge. The findings of the study are in line with the findings of prior studies (Lin, 2007c). Altruism is a trait primarily directed toward helping others with organizational tasks or problems (Organ, 1988). It encompasses the ability to feel empathy (Batson et al., 1991; De Waal, 2008), which impacts a favorable attitude to share knowledge (van den Hooff et al., 2012). Given that willingness is an attitude influenced by social capital encompassing reciprocity, ties, shared values, and language, the natural inclination of altruistic individuals leads them to rely on social capital components in their interaction with others.

Another key finding of the study is that altruism has a positive impact on tacit knowledge sharing, which is in line with previous studies (Wasko and Faraj, 2005; Wu et al., 2009). Despite the possible loss of valuable knowledge, altruistic individuals engage in knowledge sharing as they enjoy helping others. By sharing experience, stories, and information, employees contribute to the collective, possibly at their own expense. Szuster (2016) defines altruism as regulating responses to the environment through social norms. The import of selflessly achieving the “well-being of others” comes at a cost meant to invalidate suspected “egoistic” motivation for altruism. Also, given that altruism impacts willingness, which is associated with reciprocity and social ties, the mediating effect of the subjective norm on the altruism–tacit knowledge sharing relationship was confirmed. Parallel to that notion, altruistic people, possibly through their interaction and selflessness, build ties with others, which facilitates sharing of tacit knowledge. It is, therefore, confirmed that personality traits relying on social capital, such as altruism, have more influence on tacit knowledge sharing compared to personality traits that have accentuated intrinsic components.

In line with the prior studies (De Vries et al., 2006), our findings suggest that willingness is an essential factor for knowledge-sharing behavior between employees. Social capital embedded in interpersonal relationships existing among individuals is critical for facilitating the process of socialization through which willing individuals share knowledge (Huang et al., 2008). In other words, employees share knowledge to contribute to the understanding of the collective. Social interaction, shared vision, network influence, and language may contribute to shaping the subjective norm (Hu and Randel, 2014).

Social exchange theory framework was applied to create a research model that takes into account individuals’ willingness and capacity to comply with the subjective norm and share knowledge to achieve desired outcomes. It examines individual behavior as a rational social phenomenon based on the subjective cost–benefit analysis (Yan et al., 2016). Under this theory, we were able to understand the central notion of reciprocity better. Social behavior includes social exchanges where individuals are motivated to attain a reward for which they must forfeit something of value (Redmond, 2015). In this context, willingness then refers to the extent to which one is ready to communicate his/her intellectual resources and dedicate time and energy to other team or organizational members. As opposed to altruistic people, self-interested willing individuals have a low internal drive to share knowledge and should be otherwise motivated (Van den Hooff and Hendrix, 2004; Ziemba and Eisenbardt, 2014). They are, therefore, susceptible to incentives and act under the condition of reciprocity. In line with social exchange theory, willingness to share knowledge is high under the expectancy of reciprocal benefits, and it necessarily includes interpersonal trust as a form of insurance that payoffs will be received in the future (Tohidinia and Mosakhani, 2010; Krok, 2013; Kuo, 2013; Loebbecke et al., 2016). Willing individuals are prepared to take the initiative, provided that others do the same. If the applied incentives are going to motivate employees, they must be perceived as reliable, fair, and comparable with the incentives that are received by the employees’ peers. Therefore, incentives may be a significant motivational factor contributing to the frequency of knowledge contribution and transfer (Weir and Hutchings, 2005; Kim and Lee, 2006; Al-Alawi et al., 2007). Our findings are in line with those of Curtis and Taylor (2018), Kambey et al. (2018), and Le and Lei (2019).

Finally, the study confirms that subjective norm, in turn, has a positive impact on tacit knowledge sharing. Employees act according to the subjective norm and engage in an exchange of different useful forms of knowledge. The results of the study are parallel with studies applying the theory of reasoned action to explain knowledge sharing (Bock et al., 2005; Kuo and Young, 2008).

### Implications of the Study

Attaining conditions that fuel the knowledge-sharing process is a challenging endeavor. It requires developing initiatives that adequately support knowledge sharing. Such actions stem from a deep understanding of critical aspects of the sharing process, such as innate attributes of individuals. Given that personality is an indicator of knowledge-sharing behavior (Politis and Politis, 2012) and knowing that altruistic individuals are predisposed to share knowledge willingly, managers can accordingly react and set specific roles for altruistic individuals within teams. Assigning altruistic individuals, the tasks in which they would collaborate and engage in socialization with other people could encourage and enhance knowledge sharing in a group.

Additionally, to facilitate reaching desired levels of knowledge sharing, organization management should focus on identifying employees possessing the characteristic of altruism during the hiring and screening process as it is positively related to the attitude of willingness, which is relevant to knowledge sharing. By evaluating the personality traits of candidates, management develops an insight into a disposition resulting in a manner conducive to knowledge-sharing behavior. Attitude toward knowledge-sharing evaluations should be interpreted together with personality tests to minimize the risk of untruthful replies as job candidates could make themselves appear more desirable in the eyes of management by manipulating self-report questionnaires.

### Prospective Research and Limitations

Limitations of the study are characteristic of this type of empirical research. First, the triangulation method was not applied, and data were collected from a relatively small and homogeneous sample by using self-reported measures. Purposive sampling may also result in sampling bias, making the results less generalizable. We conducted our study focusing on innovation-oriented and knowledge-intensive industries, which rely heavily on the tacit knowledge of persons involved. The subjects of the study were highly educated professionals whose effective collaboration is sometimes dependent on willingness to share and engagement in tacit knowledge sharing. This may cause a specific sampling bias, which would restrict generalizability of the study. Therefore, it is recommended that future studies conduct a survey in a broader context of the general working population working across variety of industries. That said, our findings are still relevant and highly useful for knowledge-based and collaboration-dependent organizations. Furthermore, we have taken a theoretical perspective of altruism as a prosocial behavior and enjoyment in helping others in the context of knowledge sharing, and the construct may be otherwise explored depending on the definition. We, therefore, urge future empirical research to approach and conceptualize altruism from a different theoretical view, such as the constituent norm to do good, the motivation grounded in empathy, or evolutionary and hereditary characteristics.

Future studies should replicate our study in the context of one company within a single industry or on a larger sample across various sectors. Less knowledge-intensive work and projects should also be studied to examine the knowledge-sharing behaviors in such environments. Furthermore, the survey was cross-sectional with data being collected at one point in time. To validate the findings and capture the causality of the knowledge-sharing behavior, a longitudinal study is advised. Next, only a personality facet of altruism was included in the study, and others, which may be more relevant for knowledge sharing, were not a part of the study. Future studies could examine other personality traits and facets, such as consciousness, neuroticism, and competitiveness. Even though some initial insights were attained through telephone conversations conducted with employees before the survey commenced, an in-depth qualitative study could have been undertaken to surface specific individual factors conducive to knowledge sharing. Future studies could investigate how certain factors conducive to knowledge sharing, such as social capital and leadership, would interact with various personality traits to produce favorable attitudes toward knowledge sharing. Other elements that should be considered in prospective investigations are the work type characteristics, heterogeneity of team members, or dynamic work environments.

## Conclusion

The study findings contribute to the existing theory on knowledge management and organizational psychology. The altruism–willingness relationship has been examined in the context of tacit knowledge sharing. The research findings imply that altruism has a positive impact on the subjective norm and tacit knowledge sharing. The personality trait of altruism has been empirically confirmed as an antecedent of willingness to share knowledge in the current study. Another valuable contribution lies in the discovery that altruism has a direct impact on tacit knowledge sharing, reaffirming a relationship with knowledge sharing, but this time distinguishing between sharing of different types of knowledge, assessing tacit knowledge sharing as a construct separate from general knowledge sharing. Our findings suggest that willingness is an essential factor of knowledge-sharing behavior between employees, having both direct impact on tacit knowledge sharing and being a mediator between the trait of altruism and tacit knowledge sharing. Most prior studies have not conceptualized the knowledge-sharing attitude as “willingness” but have only framed it as favorable and unfavorable toward knowledge sharing. Finally, we found that the subjective norm is a mediator between altruism and tacit knowledge sharing and that it has a direct impact on tacit knowledge sharing. Existence of normative beliefs of employees concerning knowledge sharing will positively affect them to share tacit knowledge. The study contributes to the organizational psychology field and presents empirical evidence, which can be used in the development of knowledge-sharing initiatives.

## Data Availability Statement

The datasets generated for this study are available on request to the corresponding author.

## Ethics Statement

The studies involving human participants were reviewed and approved by the Ethics Committee of the Jiangsu University. Written informed consent for participation was not required for this study in accordance with the national legislation and the institutional requirements.

## Author Contributions

BO conceived the idea, contributed to the design of the study, was involved in all steps of the research process, and wrote a first setup and draft of the manuscript. DJ contributed to the design of the study, data acquisition, and result interpretation, and drafted the manuscript. DT contributed to the design of the study, data collection, and adjustments, and wrote additions. SO researched the statistical methods and contributed to analysis and result interpretation. MK made a substantial, direct, and intellectual contribution to the work, edited the manuscript, and approved it for publication. FA contributed to the statistical analysis, results interpretation, manuscript improvement, and editing. All authors contributed to the article and approved the submitted version.

## Conflict of Interest

 SO was employed by Inovatus Usluge Ltd. The remaining authors declare that the research was conducted in the absence of any commercial or financial relationships that could be construed as a potential conflict of interest.
